# Unrecognized myocardial infarction assessed by cardiac magnetic resonance imaging is associated with adverse long-term prognosis

**DOI:** 10.1371/journal.pone.0200381

**Published:** 2018-07-06

**Authors:** Anna M. Nordenskjöld, Per Hammar, Håkan Ahlström, Tomas Bjerner, Olov Duvernoy, Bertil Lindahl

**Affiliations:** 1 Department of Cardiology, Faculty of Medicine and Health, Örebro University, Örebro, Sweden; 2 Department of Radiology, Oncology and Radiation Science, Uppsala University, Uppsala, Sweden; 3 Department of Medical Sciences, Cardiology, Uppsala University, Uppsala, Sweden; 4 Uppsala Clinical Research Centre, Uppsala, Sweden; Scuola Superiore Sant'Anna, ITALY

## Abstract

**Background:**

Unrecognized myocardial infarctions (UMIs) are common. The study is an extension of a previous study, aiming to investigate the long-term (>5 year) prognostic implication of late gadolinium enhancement cardiovascular magnetic resonance (LGE-CMR) detected UMI in patients with suspected stable coronary artery disease (CAD) without previously diagnosed myocardial infarction (MI).

**Methods:**

In 235 patients with suspected stable CAD without previous MI, LGE-CMR imaging and coronary angiography were performed. LGE with a subendocardial component detectable in more than one imaging plane was required to indicate UMI. The stenosis grade of the coronary arteries was determined, including in the artery supplying an infarcted area. Stenosis ≥70% stenosis was considered significant. Patients were followed for 5.4 years in mean regarding a composite endpoint of cardiovascular death, MI, hospitalization due to heart failure, stable or unstable angina.

**Results:**

UMI were present in 58 of 235 patients (25%). Thirty-nine of the UMIs were located downstream of a significant coronary stenosis. During the follow-up 40 patients (17.0%) reached the composite endpoint. Of patients with UMI, 34.5% (20/58) reached the primary endpoint compared to 11.3% (20/177) of patients with no UMI (HR 3.7, 95% CI 2.0–6.9, p<0.001). The association between UMI and outcome remained (HR 2.3, 95% CI 1.2–4.4, p = 0.012) after adjustments for age, gender, extent of CAD and all other variables univariate associated with outcome. Sixteen (41%) of the patients with an UMI downstream of a significant stenosis reached the endpoint compared to four (21%) patients with UMI and no relation to a significant stenosis (HR 2.4, 95% CI 0.8–7.2, p = 0.12).

**Conclusion:**

The presence of UMI was independently associated with an increased risk of cardiovascular events during long-term follow up.

## Introduction

A large proportion of all acute myocardial infarctions (MIs) is not clinically recognized [1, 2]. Unrecognized myocardial infarction (UMI) is defined as a MI that is undetected during the acute phase, but eventually discovered by detection of pathological Q waves on the electrocardiogram (ECG), myocardial imaging revealing evidence of a loss of viable myocardium, or pathological findings on autopsy [1, 3]. The reason why the UMI is not recognized during the acute phase is most often unknown. Patients with UMI may not experience chest pain to the same extent as patients with clinically recognized MI or they may have other complaints not recognized as typical for MI.

The prevalence of UMI detected by ECG varies considerably depending on the cohorts studied, with a distribution between 5–44% in individuals 40–93 years of age [1, 2, 4] and between 8–36% in patients with stable coronary artery disease (CAD) [5–7]. A fairly comprehensive review has demonstrated that patients with ECG detected UMI and patients with clinically recognized MIs seems to have a similar long-term prognosis [1], however, the results are still inconsistent and a recent large study found no association between ECG detected UMI and prognosis after adjustment for traditional risk factors [8].

Late gadolinium enhancement cardiovascular magnetic resonance (LGE-CMR) imaging has improved the detection of small lesions due to MI, which do not give rise to Q-waves on the ECG [9]. Hence, the sensitivity of UMI detection with LGE-CMR is higher than with ECG [4, 6, 7, 10, 11]. The prevalence of LGE-CMR detected UMI has been reported to vary between 0.2–30% [4, 11–14] in people from the general population and between 19–27% in patients with suspected CAD [6, 7, 15–17]. The two-year prognosis in patients with suspected CAD and LGE-CMR detected UMI has been evaluated previously by us and in two other small studies; in all three studies UMI was associated with cardiovascular events and mortality in univariate analysis [6, 7, 16]. The association did however not remain statistically significant after adjustment for coexisting CAD in the only previous study adjusting for coexisting CAD [16]. Studies with long term follow-up exceeding two years are lacking.

Therefore, the purpose of this extension of our previous study [16] was to investigate the long-term (>5 year) prognostic implication of LGE-CMR detected UMI after adjustment for coexisting CAD in patients with suspected stable CAD without previously diagnosed MI.

## Methods

### Study population

The Prevalence and prognostic value of Unrecognized Myocardial Injury in stable coronary artery disease (PUMI) study is a prospective multicenter study conducted in Sweden. Details regarding the study population and the study procedures have been previously reported [15–19]. In brief, 265 patients with stable suspected CAD scheduled for elective coronary angiography were prospectively enrolled between January 2008 and March 2011. All patients were originally referred from their primary care physician to an internal medicine/cardiology clinic due to symptoms suggestive of stable angina pectoris. Enrollment in the present study was only considered after the patient’s history and previous examinations was re-assessed by a cardiologist at respective clinic and the patient was scheduled for elective coronary angiography. Patients with pathological Q-wave in ECG, kidney failure (estimated glomerular filtration rate (GFR) < 30 ml/min/1,73m2), history of previous MI, coronary interventions or heart failure were excluded. The LGE-CMR imaging was performed at a median of four days after enrolment (inter quartile range (IQR) 0–11 days) and the elective coronary angiography was performed at a median of nine days (IQR 7–15 days) further thereafter.

**Fig 1:**
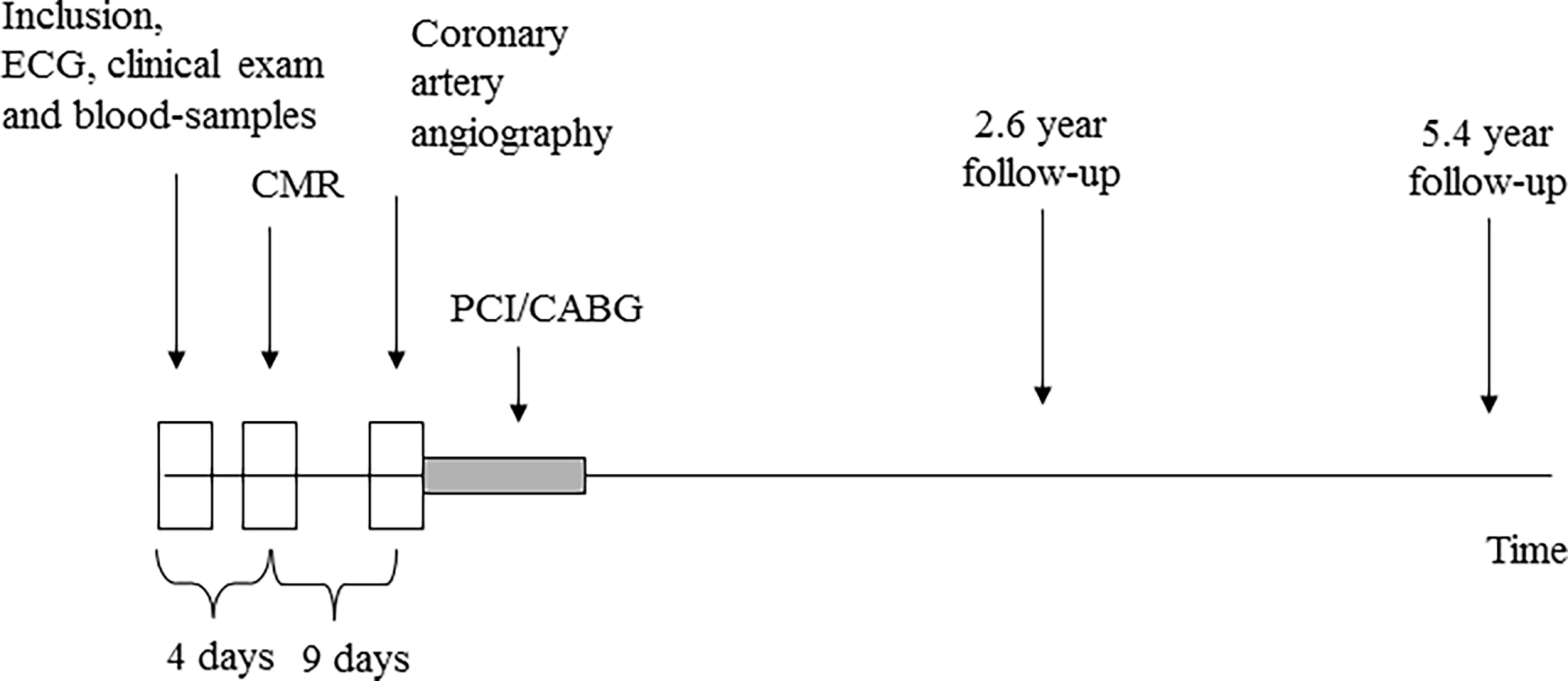
Study outline.

The 235 patients with a CMR investigation and a coronary angiography of technically adequate quality to enable analysis continued formed the cohort of the present study. The study procedure is outlined in Fig 1.

The Ethical Review Board in Uppsala, Sweden (Dnr 2007/214) approved the study. The study was registered at ClinicalTrials.gov (NCT01257282). Written informed consent was obtained from all patients.

#### Outcome definitions

Our pre-specified primary endpoint in our previous two-year follow-up report was a composite of death, resuscitated cardiac arrest, MI and hospitalization for heart failure or unstable angina [16]. However, the primary endpoint for the five-year follow-up was adjusted to a composite of cardiovascular death, resuscitated cardiac arrest, MI, hospitalization for angina pectoris and heart failure. There are two reasons for this change: first, in long-term follow-up the proportion of non-cardiovascular deaths increases by time and only the association between cardiovascular death and UMI seems relevant for the assessment of prognostic implication of UMI; second, the validity of the division in hospitalized patients in stable angina pectoris and unstable angina pectoris as primary diagnosis in the National Patient register is uncertain. Therefore, we used the joint diagnostic code for angina pectoris, International Classification of Diseases-10 code (ICD) I.20. Events were registered as first occurring.

### Follow-up

All patients were monitored from admission in the Swedish population register, National Patient register and Causes of Death register until death or the end of December 2015 (mean follow-up 5.4 years).

Follow-up data became available by merging data from the mandatory Swedish Cause of Death Register and the National Patient Register with the present study database. The merging was performed at the National Board of Health and Welfare in Sweden based on the personal identification number that all Swedish citizens and all permanent residents of Sweden have.

Data on death was obtained from the Cause of Death Register, cardiovascular death was defined as ICD I00-I78. Data on myocardial infarction (I21-I23), heart failure (I50, K761, I971, I110), ischemic stroke (I63, I64) and angina pectoris (I20) were obtained from the National Patient register, which includes ICD codes from all hospital admissions in Sweden.

### Electrocardiogram

A 12-lead resting ECG was obtained at inclusion from all patients and ECG changes were classified according to the Minnesota Code Classification System for Electrocardiographic Findings [20].

### Cardiac magnetic resonance and coronary angiography

Acquisition and analysis of CMR image investigations and coronary angiographies have previously been described in detail [15–17]. The American Heart Association model [21] was used to devide the myocardium into 17 different segments. Two radiologists (P.H and T.B) independently reviewed the CMR images for areas of late gadolinium enhancement (LGE) detectable in more than one imaging plane. A subendocaridal component of LGE was required to indicate UMI. Only information regarding left ventricle ejection fraction (LVEF) and wall motion abnormalities were made known to the physician in charge.

All coronary angiographies were analyzed independently by two radiologists (P.H and O.D) unaware of the results of LGE-CMR. The 16 segment model by Austen [22] was used to devide the coronary arteries into a total of 19 segments. The degree of diameter stenosis was categorized visually as 0–29%, 30–49%, 50–69%, 70–99% or 100%. We visually assessed which of the myocardial segments in the 17-segment model [21] that were supplied by stenoses of ≥30%. Stenoses ≥70% was considered significant.

The extent of atherosclerosis was determined by the number of vessels affected by a ≥70% stenosis.

### Statistical analyses

All analyses were pre-specified in the clinical study protocol except one post-hoc analyse regarding the relationship between UMI size and prognosis. Not normally distributed continuous variables are presented as median and inter quartile range (IQR). The Mann Whitney U-test was used for comparison used for not normally distributed data. The categorical variables are presented as frequency values and comparisons were made using either Chi-square tests or Fisher´s exact test. Age was analyzed both as a continuous variable and as a categorical variable. UMI, gender, degree of CAD and extent of CAD were analyzed as categorical variables. In order to identify the clinical characteristics associated with UMI and the relationship with the primary endpoint, univariate and multivariate Cox regression analyses were performed. All factors univariate associated with the outcome (UMI, hypertension, NT-proBNP >125 ng/L, extent of CAD) together with age and gender were assessed by multivariable cox regression. The assumption of proportional hazards was verified by Schoenfeld residual test and time-varying covariates have been assessed. Results are presented as Hazard ratios (HR) with 95% confidence intervals (CIs). All statistical tests were two-tailed and p<0.05 was regarded as statistically significant.

Data analyses were performed using the SAS (version 9.4; SAS Institute, Cary, North Carolina) or the Predictive Analytical SoftWare (PASW statistics 17.03) program (SPSS Inc, Chicago, IL, USA).

## Results

### Clinical characteristics and prevalence of UMI

**Fig 2:**
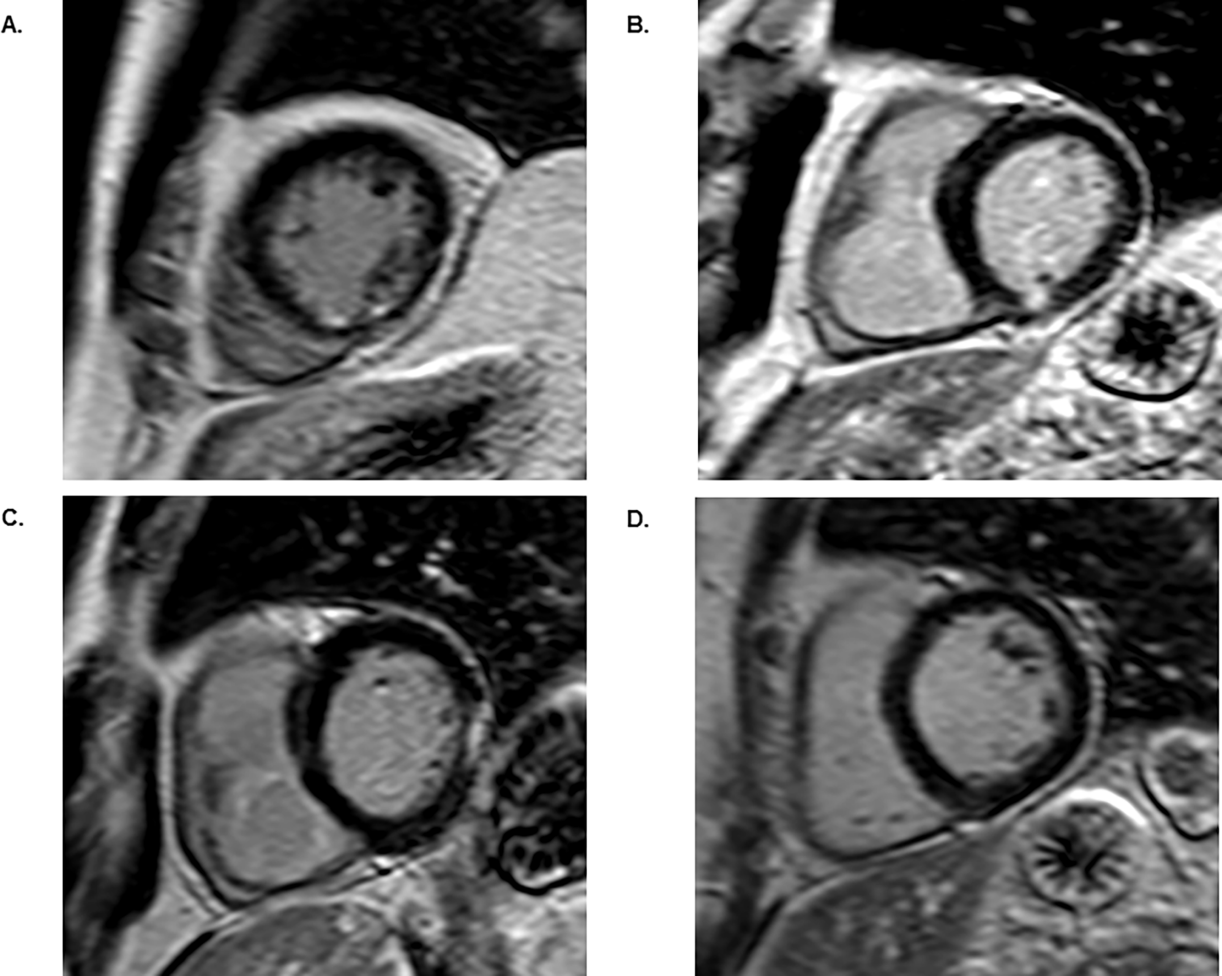
Examples of representative CMR-LGE images in patients with and without UMIs. **a**. subendocardial UMI, **b**. transmural UMI, **c.** area of LGE without a subendocardial component, i-e. no MI, **d.** no LGE, i.e. normal myocardium.

The clinical characteristics and findings at coronary angiography of the study population are shown in Table 1. As previously reported 25% (58/235) of the patients were affected by UMI [15–17]. Two thirds of the UMIs was located downstream of a significant stenos/occlusion and one third of the UMIs was not related to a significant stenosis [15]. Four patients had wall motion abnormalities assessed by CMR. All four patients had an UMI and three had also obstructive CAD, although only two exhibited an UMI in an area supplied by an artery with a significant stenosis. Examples of representative CMR-LGE images in patients with and without UMIs are shown in Fig 2.

**Table 1:** Clinical characteristics.

Characteristic	All patients	No UMI	UMI	P-value
Number	235	177	58	
Age, years, median (IQR)	65 (60–71)	65 (60–70)	66 (64–72)	0.06
Women (%)	80 (34)	66 (37)	14 (24)	0.08
LVEF, median (IQR)	66 (62–72)	66 (61–71)	67 (62–72)	0.43
NT-proBNP ng/L, median (IQR)	102(52–204)	94 (48–156)	173(65–263)	0.006
**CAD risk factors**				
Waist, cm, median (IQR)	100 (93–107)	99 (92–106)	103 (95–109)	**0.04**
Family history of CAD (%)	117 (50)	87 (49)	30 (52)	0.76
Previous or present smoking (%)	143 (61)	105 (59)	38 (66)	0.40
Hypertension (%)	132 (56)	94 (53)	38 (66)	0.13
Diabetes mellitus (%)	49 (21)	32 (18)	17 (29)	0.09
**Coronary angiography**				
Stenosis >70% (%)	135 (57)	88 (50)	47 (81)	**<0.001**
Three vessel disease (%)	23 (10)	9 (5)	14 (24)	**<0.001**
Stenosis <50% (%)	92(39)	82 (46)	10 (17)	**<0.001**
**Revascularized after angiography**				
PCI (%)	98 (42)	61 (34)	37 (64)	**<0.001**
CABG (%)	23 (10)	15 (8)	8 (14)	0.42

CABG; Coronary Artery Bypass Grafting, CAD; Coronary Artery Disease, IQR; inter-quartile range, PCI; Percutaneous Coronary intervention, UMI; Unrecognized Myocardial Infarction.

### UMI and prognosis

During the total follow-up period of in mean 5.4 years (mean 65, minimum 1 and maximum 95 months), 83 adverse events occurred in 47 patients. No patient was lost to follow-up.

A total of 15 (6.4%) patients died and the cause of death were cardiovascular in seven (46.7%) patients, cancer in five patients and pulmonary in three patients. Death occurred in five patients with UMI and 10 patients without UMI (p = 0.42). Cardiovascular death affected three patients with UMI and four patients without UMI (p = 0.37).

A total of 18 MIs occurred in 14 patients, 11 in patients with UMI and 7 in patients without UMI (p = 0.06). There were 43 hospitalizations in 31 patients due to instable och stable angina pectoris, 23 in patients with UMI and 20 in patients without UMI (p<0.001). Hospitalization due to heart failure affected three patients once, one with UMI and two without UMI (p = 1.00). Six hospitalizations due to other heart disease affected four patients, three with UMI and three without UMI (p = 0.06). No patient suffered a resuscitated cardiac arrest.

Forty patients (17.0%) reached the composite endpoint (four cardiovascular deaths, 10 MIs, 23 hospitalizations for angina pectoris and three for heart failure).

**Fig 3:**
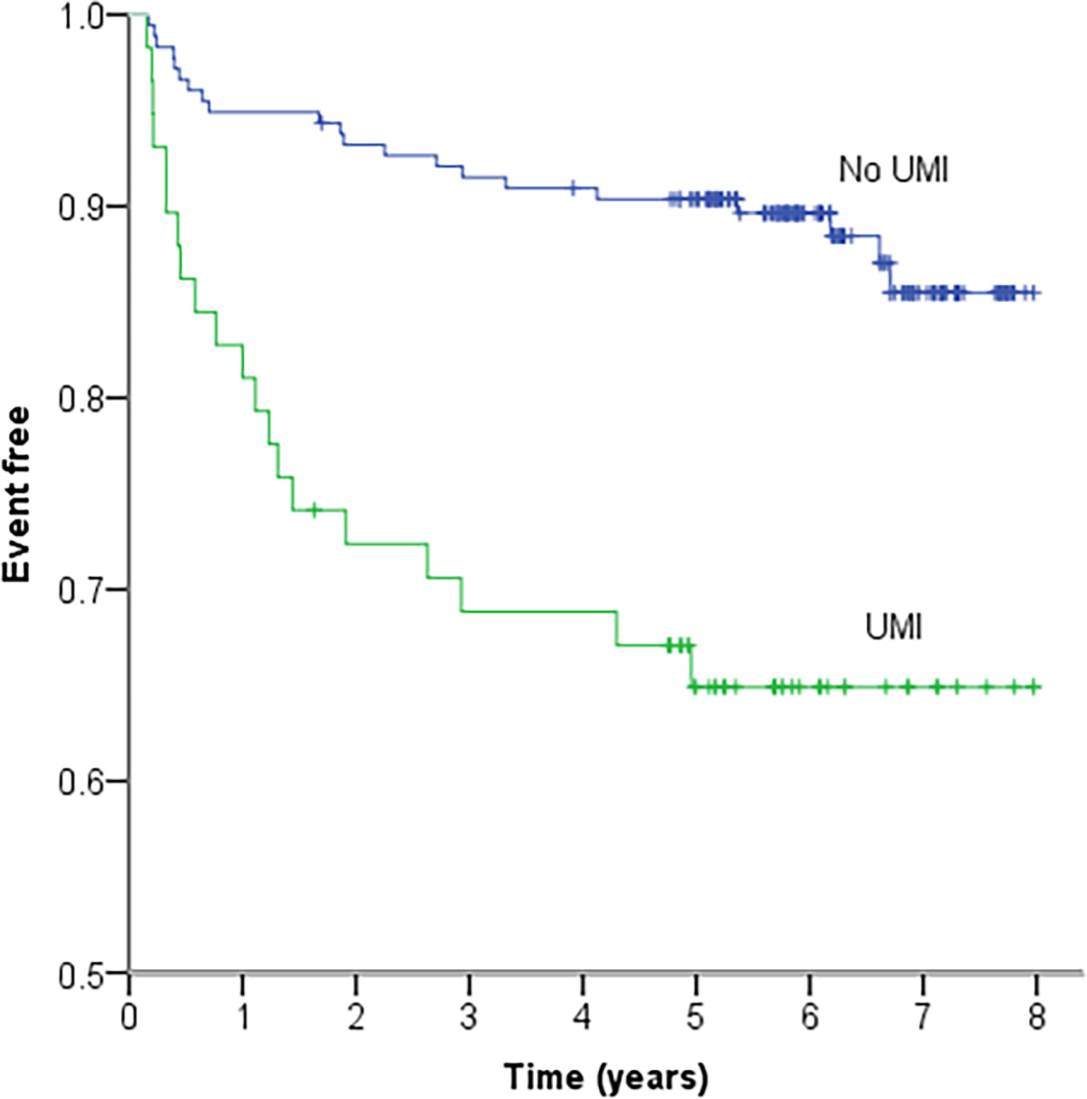
Kaplan-Meier plot of the cumulative probability of remaining event free for patients with UMI versus patients without UMI. P<0.001. Blue line = No UMI. Green line = UMI.

A total of 34.5% (20/58) of patients with UMI reached the composite endpoint compared to 11.3% (20/177) of patients with no UMI (HR 3.7, 95% CI 2.0–6.9, p<0.001) (Table 2 and Fig 3). Besides UMI, hypertension, NT-proBNP >125 ng/L, the presence of a significant coronary artery stenosis and the extent of CAD were significant univariate predictors of outcome (Table 2).

**Table 2:** Univariate predictors of prognosis.

Characteristic		Endpoint	HR (95% CI)	P-value
**Age**	≤Median age 65.4 years (no = 117)	16.2%		
** **	>Median age 65.4 years (no = 118)	17.8%	1.1 (0.6–2.1)	0.682
**BMI**	<25 (no = 60)	13.3%		
** **	≥25 (no = 175)	18.2%	1.4 (0.7–3.1)	0.386
**Diabetes**	No (no = 186)	15.1%		
** **	Yes (no = 49)	24.5%	1.7 (0.9–3.4)	0.111
**Gender**	Women (no = 80)	12.5%		
** **	Male (no = 155)	19.4%	1.6 (0.8–3.3)	0.198
**Hypertension**	No (no = 103)	9.7%		
** **	Yes (no = 132)	22.7%	2.5 (1.2–5.2)	**0.011**
**LVEF**	<50% (no = 9)	3.8%		
** **	≥50% (no = 102)	96.2%	0.7 (0.1–3.1)	0.680
**Smoking**	Never (no = 92)	16.3%		
** **	Previous or present (no = 143)	17.5%	1.1 (0.6–2.0)	0.825
***Biochemistry***				
**Hs-cTnI**	≤ 5ng/L (no = 151)	14.6%		
** **	5–23 ng/L (no = 72)	22.2%	1.6 (0.9–3.1)	0.131
** **	≥23 ng/L (no = 12)	16.7%	1.1 (0.3–4.8)	0.875
**NT-proBNP**	≤ 125ng/L (no = 133)	10.5%		
** **	> 125ng/L (no = 102)	25.5%	2.7 (1.4–5.1)	**0.003**
***Investigations***				
**CMR image**	Without UMI (no = 177)	11.3%		
** **	With UMI (no = 58)	34.5%	3.7 (2.0–6.9)	**<0.001**
**Coronary angiography**	No significant coronary stenosis(no = 135)	4.0%		
	Significant coronary stenosis (no = 100)	26.7%	7.6 (2.7–21.4)	**<0.001**
	Extent of coronary artery disease			
	No ≥70% stenosis (no = 100)	4.0%		
	One vessel disease (no = 67)	20.9%	5.6 (1.9–7.1)	**0.002**
	Two vessel disease (no = 45)	33.3%	10.3(3.4–1.1)	**<0.001**
	Three vessel disease (no = 23)	30.4%	9.1 (2.7–31.0)	**<0.001**

BMI; body mass index, CMR; cardiac magnetic resonance, LVEF; left ventricular ejection fraction, UMI; unrecognized myocardial infarction.

The UMIs were transmural in 21 patients, and subendocardial in 37 patients. Transmural and subendocardial UMI were both univariate associated with the outcome. In a multivariable Cox analysis both had the same independent predictive value; subendocardial UMI HR 2.3 (95% CI 1.1–4.8) and transmural UMI HR 2.6 (95% CI 1.1–6.4).

The median UMI size was 2.2 mL and 31% (9/29) of the patients with a UMI size below median and 38% (11/29) of the patients with a UMI size above median reached the composite endpoint (p = 0.58).

All factors univariate associated with the outcome together with age and gender were assessed by multivariable logistic regression (Table 3). The presence of UMI, NT-proBNP >125 ng/L and the extent of CAD remained statistically significantly associated with outcome.

**Table 3:** Multivariable logistic regression analyses of the primary endpoint.

Covariat	HR (95% CI)	P-value
**Age**	1.5 (0.8–3.0)	0.203
**Gender**	1.0 (0.5–2.0)	0.930
**Hypertension**	1.9 (0.9–3.9)	0.081
**NT-proBNP >125ng/L**	2.3 (1.1–4.5)	**0.019**
**UMI**	2.3 (1.2–4.4)	**0.012**
**Extent of CAD**		
**No ≥70% stenosis**	reference	
**One vessel disease**	5.0 1.6–15.6)	**0.005**
**Two vessel disease**	8.1 (2.6–25.4)	**<0.001**
**Three vessel disease**	5.5 (1.5–19.8)	**0.010**

CAD; Coronary artery disease, UMI; unrecognized myocardial infarction.

A total of 41% (16/39) of the patients with an UMI located downstream of a significant stenos/occlusion reached the endpoint compared to 21% (4/19) in patients with UMI and no relation to a significant stenosis (HR 2.4, 95% CI 0.8–7.2, p = 0.12).

#### Discussion

In the present study LGE-CMR detected UMIs were associated with more than a threefold increased risk of the composite endpoint during the five year follow-up, as many as 34.5% of patients with UMI, compared to 11.3% of patients without UMI, suffered adverse cardiovascular events. The association was mainly driven by the increased number of hospitalizations for angina in patients with UMI. The association remained even after adjustment for traditionally risk factors and the severity of CAD, thereby extending the findings of an association between UMI and future cardiac events in previous short-term studies of patients with suspected or confirmed stable CAD [6, 7, 23] and in general populations [4, 24]. A recent study of patients with a first clinical episode of MI and a LGE-CMR detected UMI in a non-culprit territory, also demonstrated an independent association between UMI and prognosis, whereas, in contrast to our study, angiography-proven multi-vessel disease did not [25].

The event rate among stable CAD patients with UMI in the present study was considerably higher than the 11-year composite event rate of 20% seen in individuals with LGE-CMR detected UMI from the general population [24]. The average annual risk for an adverse cardiovascular event was 3.2% in our study population, which is in accordance with a recent report based on patients with stable CAD in two different cohorts where the annual risk for cardiovascular events (MI, stroke or cardiovascular death) were 2.2 and 3.4%, respectively [26]. This indicates that our study cohort is representative for patients with stable CAD. In contrast, the average annual mortality rate of 1.3% in the present study was considerably lower than the annual mortality rate of 11% and 22%, respectively, in two previous UMI studies [6, 7]. The discrepancy in mortality between these previous UMI studies and ours is probably due to differences in selection, characteristics and/or treatment of the patients.

### ECG detected UMI versus LGE-CMR detected UMI

The prevalence and prognostic value differ depending both on whether ECG or LGE-CMR is used for diagnosis of UMI. There are several ECG related issues to consider; first, the accepted ECG criteria for UMI have changed over time [1, 3]; secondly, not all MIs result in pathological Q waves [27]; thirdly, several cardiac and non-cardiac conditions can produce ECG changes mimicking those associated with MI and confound the diagnosis [3] and fourthly, ECG may change over time, and a pathological Q-wave can disappear [28]. It has been estimated that ECG features of MI disappear within two years in 10% of subjects with anterior MI and in 25% of those with an inferior MI [29]. In a recent study, opportunistic identification of asymptomatic Q-waves by routine ECG were demonstrated to clearly overestimate UMI (detected with different imaging technics as gold standard), especially in those with at low cardiovascular risk [30]. Therefore, a comparison between the prevalence and prognostic value of ECG-detected and LGE-CMR-detected UMIs is difficult. In our cohort, all subjects with Q-waves on ECG were excluded. Hence, none of the UMIs in the present study would have been detected as UMI in an ECG-study.

### Pathophysiology of UMI and prognosis

The pathophysiological mechanisms behind UMI are poorly defined. However, we and others have previously been able to describe a significant association between UMI and the severity and extent of CAD [6, 15] and that as many as two thirds of the UMIs are directly downstream of a severe stenosis or occlusion [15]. The atherosclerotic process may include silent plaque rupture, ulceration, fissuring, erosion and thrombosis [31, 32], which might occasionally cause UMI. Furthermore, a severe coronary stenosis increases the risk for ischemic myocardial injury due to supply/demand mismatch. However, one sixth of the UMIs in the present study population occurred in patients without any significant CAD [15, 16], which indicates that other underlying pathophysiological mechanisms than CAD may cause UMI.

There was no significant difference in outcome between patients with or without UMI downstream of a significant stenos/occlusion in the present study, although the event rate was numerically higher in patients with an UMI downstream a significant coronary stenosis. However, due to the low number of events the power to detect a significant difference was low.

Another unresolved issue is why the myocardial injury, irrespectively of the underlying cause, is undetected in patients with UMI. Myocardial ischemia stimulates free nerve endings, which usually gives rise to the conscious perception of chest pain [28]. One possible mechanism for the lack of obvious symptoms of AMI may be increased pain tolerance, which has been described in UMI patients [33]. However, all patients in the present study had symptoms suggestive of stable angina pectoris for which they have sought medical attention; and hence, were able to perceive chest pain. Nevertheless, regardless of the mechanisms involved, failure to recognize myocardial ischemia and hence receive adequate therapy may in itself increase the risk of experiencing new cardiac events and complications.

### Clinical implications

It is unknown if the prognosis is possible to improve by any specific treatment in patients with UMI. Therefore, although a LGE-CMR detected UMI is independently associated with an adverse prognosis, we do not think that the results support routine investigation by DE-CMR in patients with suspicion of CAD. However, it seems reasonable to optimize treatment of cardiovascular risk factors as well as consider coronary angiography in patients with cardiac symptoms, in patients with en passant detected UMI at cardiac DE-CMR.

### Limitations

There are some limitations to the present study that need to be considered. First, the study cohort consists of patients with suspicion of stable angina pectoris and generalizations of the results to other groups should be done with caution. Second, the number of events was low giving limited power to demonstrate significant associations. However, the present study included more patients than previous studies [6, 7] and the follow-up time was considerably longer than in other studies [6, 7, 16, 23]. Third, different CMR scanners were used at the different sites and the examinations protocols were therefore not exactly the same for all examinations. However, all LGE-CMR images were centrally reviewed independently by two radiologists. Fourth, all coronary angiographies were performed locally and all decisions regarding coronary interventions and medications were made by the local physician in charge. An additional examination such as fractional flow reserve may have been performed in some cases, but no such information was passed on to the present study. However, all coronary angiographies were reviewed centrally by two independent examiners. Fifth, the follow-up data became available by merging data from the mandatory Swedish Cause of Death Register and the National Patient Register with the present study database. The diagnoses used are discharge diagnoses (ICD-10 codes) made in clinical routine and thus probably not as accurate as if they have been centrally adjudicated by a group of experts. However, primary diagnoses of MI, stroke, and heart failure in the Swedish National Patient Register have all been shown to have high validity [34–36]. Because of the unique Swedish personal identification number, we were able to get complete follow-up in all patients.
